# Case of Supposed Violence or Poisoning

**Published:** 1851-05

**Authors:** E. C. Banks

**Affiliations:** Charlestown, Illinois


ARTICLE VII.

*Case of supposed Violence or Poisoning—Post Mortem Examination.* By E. C. Banks, M. D., of Charlestown, Illinois.

I was called on, a few days since, to make, with Dr. T——, a post mortem examination in a case of death, supposed to be by injury or poisoning, of a woman aged about fifty years, and who had been dead ten and buried eight days. The circumstances are briefly these, as near as I could gather from all parties: The woman was frequently seized with a paroxysm of supposed cholic after straining much on lifting anything; rupture was discovered and the fact communicated to her husband and a few neighbor women several years since; but when the spells came on her they were always the result of violent straining, and would only cease after she herself adjusted the tumor by pressing her fingers thereon, and in that way reduce the hernia. Her husband being an ignorant fellow, and more like a brute than a civilized man, never consulted any one concerning her situation, and she never wore a truss in her life. He says (and the information communicated by him and these women, though exceedingly vague and unsatisfactory, is, nevertheless, all we possess up to the *post mortem*), the rupture was only occasionally of any inconvenience, and only became painful after the lifting and straining above alluded to. The following testimony, with the above, was elicited from her husband and the women:

Her husband stated, after being arrested, that his wife had been doing something that brought on one of these paroxysms, and that she grew so bad he wished and proposed going after some of the neighboring women, but she would not consent, saying that she would soon be better. She remained a few hours in this condition (time not stated), and, evening having drawn on, he and son went out to do the chores, and left her alone. When they came in he discovered great alteration in her symptoms, and on going to the bed found her speechless; went for neighboring women himself, and when he returned she was dead, and the women say cold and stiff, except her neck, which they thought wore marks of violence, finger prints, and they thought her neck broken simply because it was limber. They dressed her and saw no other marks of violence, and hence rumor soon spread that violence inflicted by her husband caused her death. Still she was buried and remained interred eight days, when the rumor had become so strong that a Coroner's inquest was called and the body disinterred.

One reason for supposing he killed her, was the disagreeable life they were known to have lead; he frequently beating her and driving her out at mid-night, in a cold storm, when she would be compelled to take refuge in some neighbor's house. This, with the sudden death and supposed marks of violence, all contributed to confirm in the minds of his neighbors the belief of foul usage causing her death. On inspecting the brain no marks of injury appeared; the scalp sound, neck not dislocated, no marks of violence on the throat, trachea healthy as far as possible to determine, lungs and heart sound, veins no more than usually filled with blood, liver and stomach sound, no scum in the chest or abdomen. But on inspecting the bowels, that portion of the colon coming off first from the cœcum and a few inches therefrom, was found to be immovable to any slight traction, and on making more powerful traction, the intestine, highly diseased and engorged, slipped from the sack formed in the groin by the passage of the intestine through the inguinal ring, and the enlargement of the sack was considerable. The inguinal ring was enlarged to the size of a man's thumb, and the sack would have nearly contained an ordinary fist. The intestine was perfectly impacked with fecal matter, and strangulation was the result. The entire colon gave some evidence of disease even at that date; and that portion contained within the sack was highly infected and soft, while the internal lining of the sack was also greatly diseased. The soft and impacked condition of the intestine, and the condition of the sack and entire colon, led us to determine that the woman came to her death from strangulated hernia, in the absence of any other visible cause to account for her death.

